# Discriminative Features in Three Autosomal Recessive Cutis Laxa Syndromes: Cutis Laxa IIA, Cutis Laxa IIB, and Geroderma Osteoplastica

**DOI:** 10.3390/ijms18030635

**Published:** 2017-03-15

**Authors:** Ariana Kariminejad, Fariba Afroozan, Bita Bozorgmehr, Alireza Ghanadan, Susan Akbaroghli, Hamid Reza Khorram Khorshid, Faezeh Mojahedi, Aria Setoodeh, Abigail Loh, Yu Xuan Tan, Nathalie Escande-Beillard, Fransiska Malfait, Bruno Reversade, Thatjana Gardeitchik, Eva Morava

**Affiliations:** 1 Kariminejad-Najmabadi Pathology & Genetics Center, #2, 4th Street, Hasan Seyf Street, Sanat Square, Tehran 14667-13713, Iran; afroozan@yahoo.com (F.A.); b_bzwr@yahoo.com (B.B.); 2 Department of Dermatopathology, Razi Dermatology Hospital, Tehran University of Medical Sciences, Tehran 14167-53955, Iran; dermpath101@gmail.com; 3 Department of Pathology, Cancer Institute, Imam Khomeini Hospital Complex, Tehran University of Medical Sciences, Tehran 14197-33141, Iran; 4 Clinical Genetics Division, Mofid Children’s Hospital, Faculty of Medicine, Shahid Beheshti University of Medical Sciences, Tehran 15514-15468, Iran; susanakbaroghli@yahoo.com; 5 Genetic Research Centre, University of Social Welfare and Rehabilitation Sciences, Tehran 19857-13834, Iran; hrkhkh@yahoo.com; 6 Mashhad Medical Genetic Counseling Center, Social Welfare and Rehabilitation Organization, Mashhad 91767-61999, Iran; mojahedi1381@yahoo.com; 7 Division of Pediatric Endocrinology and Inherited Metabolic Disorders, Department of Pediatrics, Tehran University of Medical Sciences, Tehran 14197-33141, Iran; arset59@yahoo.com; 8 Institute of Medical Biology, A*STAR, Singapore 138648, Singapore; abigail.loh@reversade.com (A.L.); t_yuxuan@yahoo.com.sg (Y.X.T.); nathalie.escande@reversade.com (N.E.-B.); bruno@reversade.com (B.R.); 9 Center for Metabolic Diseases, Department of Pediatrics, University Hospitals Leuven, Leuven 3000, Belgium; Fransiska.Malfait@UGent.be; 10 Department of Pediatrics, Radboud University Nijmegen Medical Center, Nijmegen, Gelderland 9102-6500, The Netherlands; Thatjana.Gardeitchik@radboudumc.nl (T.G.); emoravakozicz@tulane.edu (E.M.); 11 Hayward Genetics Center, Tulane University Medical School, New Orleans, LA 70112, USA

**Keywords:** autosomal recessive cutis laxa 2A, autosomal recessive cutis laxa 2B, geroderma osteodysplastica, Pyrroline-5-carboxylate reductase 1 (PYCR1), ATPase, H+ transporting lysosomal V0 subunit A2 (ATP6V0A2), GOLGIN, RAB6-INTERACTING (GORAB)

## Abstract

Cutis laxa is a heterogeneous condition characterized by redundant, sagging, inelastic, and wrinkled skin. The inherited forms of this disease are rare and can have autosomal dominant, autosomal recessive, or X-linked inheritance. Three of the autosomal recessive cutis laxa syndromes, namely cutis laxa IIA (ARCL2A), cutis laxa IIB (ARCL2B), and geroderma osteodysplastica (GO), have very similar clinical features, complicating accurate diagnosis. Individuals with these conditions often present with cutis laxa, progeroid features, and hyperextensible joints. These conditions also share additional features, such as short stature, hypotonia, and congenital hip dislocation, but the severity and frequency of these findings are variable in each of these cutis laxa syndromes. The characteristic features for ARCL2A are abnormal isoelectric focusing and facial features, including downslanting palpebral fissures and a long philtrum. Rather, the clinical phenotype of ARCL2B includes severe wrinkling of the dorsum of the hands and feet, wormian bones, athetoid movements, lipodystrophy, cataract and corneal clouding, a thin triangular face, and a pinched nose. Normal cognition and osteopenia leading to pathological fractures, maxillary hypoplasia, and oblique furrowing from the outer canthus to the lateral border of the supraorbital ridge are discriminative features for GO. Here we present 10 Iranian patients who were initially diagnosed clinically using the respective features of each cutis laxa syndrome. Each patient’s clinical diagnosis was then confirmed with molecular investigation of the responsible gene. Review of the clinical features from the cases reported from the literature also supports our conclusions.

## 1. Introduction

Cutis laxa is a heterogeneous condition characterized by redundant, sagging, inelastic, and wrinkled skin. The many forms of this disorder may have autosomal dominant, autosomal recessive, or X-linked inheritance. The autosomal recessive cutis laxa conditions have been categorized into types I, II, and III. Autosomal recessive cutis laxa type I ARCL1; Online Mendelian Inheritance in Man (OMIM#219000) is characterized by systemic involvement with respiratory, cardiovascular, and gastrointestinal manifestations. It is further divided into types IA (ARCL1A, OMIM#219100), IB (ARCL1B, OMIM#614437), and IC (ARCL1C, OMIM# 613177) caused by mutations in the Fibulin 5 (*FBLN5*), EGF-CONTAINING FIBULIN-LIKE EXTRA CELLULAR MATRIX PROTEIN 2 (*EFEMP2*) (formerly fibulin-4 FBLN4), and LATENT TRANFORMING GROWTH FACTO_BETA_BINDING PROTEIN 4 (*LTBP4*) genes, respectively. Type II is divided into type IIA (ARCL2A, OMIM#219200) and IIB (ARCL2B, OMIM#612940) caused by abnormal *ATP6V0A2* and *PYCR1* genes, respectively [1,2,3] Type III is also divided into two types, type IIIA (*ARCL3A*, OMIM#219150) and IIIB (ARCL3B, OMIM#614438), caused by mutations in ALDEHYDE DEHYDROGENASE 18 FAMILY, MEMBER A1 (*ALDH18A1*) and *PYCR1* genes respectively. Geroderma osteodysplastica (GO, OMIM#231070), another subtype of cutis laxa not included in this classification, is caused by recessive mutations in the *GORAB* gene. Whereas macrocephaly, alopecia, cutis laxa, and scoliosis syndrome (MACS, OMIM 613075) is caused by mutations in *RIN2*.

ARCL2A, ARCL2B, and GO manifest with many of the same features, making a definitive clinical diagnosis difficult. ARCL2A is characterized by generalized cutis laxa, persistent open fontanel, oxycephaly, and hyperextensible joints, with varying neurological, facial, and congenital abnormalities. Neurological findings typically observed include developmental delay, intellectual disability, hypotonia, microcephaly, hearing loss, seizures, and a cobble-like dysgenesis demonstrated by MRI of the brain [4,5,6]. Eye abnormalities, such as myopia and strabismus, may also be present. Facial dysmorphic features include frontal bossing, reverse V eyebrows, downslanting palpebral fissures, a long philtrum, and sagging cheeks with anteverted nares. Additional features that may be observed are intra-uterine growth retardation, blue sclera, pectus excavatum, inguinal hernia, flat feet, and congenital hip dislocation [7,8]. Biochemical analysis revealed that these patients have a combined defect of N- and O-glycosylation of serum proteins [1,4,9]. Affected individuals show a reduction in the isoeclectric focusing main protein band, corresponding to transferrin containing four sialic acid residues and increased amounts of disialo- and trisialo-transferrin that indicate altered N glycosylation over the normal range [4,9,10]. Serum apolipoprotein C III isoelectric focusing also reveals reduction of apolipoprotein CIII containing two sialic acid residues and increased amounts of monosialotransferrin. Infants may have a normal transferrin isofocusing profile in the first months of their lives but, if repeated later on, will develop the typical transferrin abnormality [9,10]. Biochemical analysis is good for identification of ARCL2A patients but it cannot be used for identification of ARCL2B and GO cases.

ARCL2B is characterized by intra-uterine growth retardation, cutis laxa, wrinkling of skin, particularly of dorsum of hands and feet, visible veins on the chest, congenital hip dislocation, hyperextensible joints, and adducted thumbs. Phenotypic facial features include a broad and prominent forehead, aged appearance, triangular face, and thin nose [11,12,13,14]. Neurological findings observed in this condition are hypotonia, developmental delay, intellectual disability, and dysgenesis or agenesis of the corpus callosum [11]. Normal intelligence has been reported in a few patients [15,16,17]. Some patients also demonstrate lipodystrophy, osteoporosis/osteopenia, and blue sclera. Biallelic *PYCR1* mutations were identified in patients previously diagnosed with GO, wrinkly skin syndrome, or ARCL2 [2,3], but these mutations are now classified as ARCL2B.

GO was first described by Bamatter et al. in five members from a Swiss family [18]. This condition is characterized by premature aged appearance, drooping cheeks, maxillary hypoplasia, wrinkled skin, and osteopenia [15]. There is also an oblique furrowing extending from the outer canthus to the lateral border of the supraorbital ridge [19]. Due to the osteopenia, the bones, particularly the vertebrae, are susceptible to fracture [20]. Individuals affected by GO do not have any intellectual disability, but they may develop congenital hip dislocations, hypotonia and short stature. Hennies et al. identified recessive mutations in the *GORAB* gene in patients diagnosed with GO [21].

Rajab et al. reported on 22 patients with the diagnosis of wrinkly skin syndrome or GO and concluded that these are two distinct disorders. Genetic testing performed on many patients diagnosed with wrinkly skin syndrome showed mutations in *ATP6V0A2* or *PYCR1* [1,2].

## 2. Patients

An in depth clinical evaluation, with documentation of phenotypic features, was completed for each patient. Detailed clinical data was obtained by a standardized questionnaire. All families provided written informed consent according to institutional guidelines. Ethics approval was obtained by the Kariminejad-Najmabadi Pathology and Genetics Center ethical committee.

The observed characteristics and symptoms were then compared against the discriminative features of each cutis laxa syndrome to preliminarily diagnose each patient. The clinical diagnoses indicated which genes needed to investigated by Sanger sequencing in each case. In all cases the candidate genes suggested by the clinical phenotype were then confirmed by molecular investigation.

Here we report 10 patients with ARCL2A, ARCL2B, or geroderma osteodysplastic (GO) and discuss the clinical features that help differentiate between these three cutis laxa syndromes. The clinical findings from each patient are reported in Table 1. Table 2 is a comparison of the frequency of the clinical features observed in ARCL2A, ARCL2B, and GO as reported in the literature against the frequency in our cases (Table A1, Table A2 and Table A3).

## 3. Discussion

Although there is a large phenotypic overlap among the three types of cutis laxa syndromes, ARCL2A, ARCL2B, and GO, in this manuscript we highlight the main differences that allow for clinical discrimination amongst them.

### 3.1. Overlapping Features

**Figure 1:**
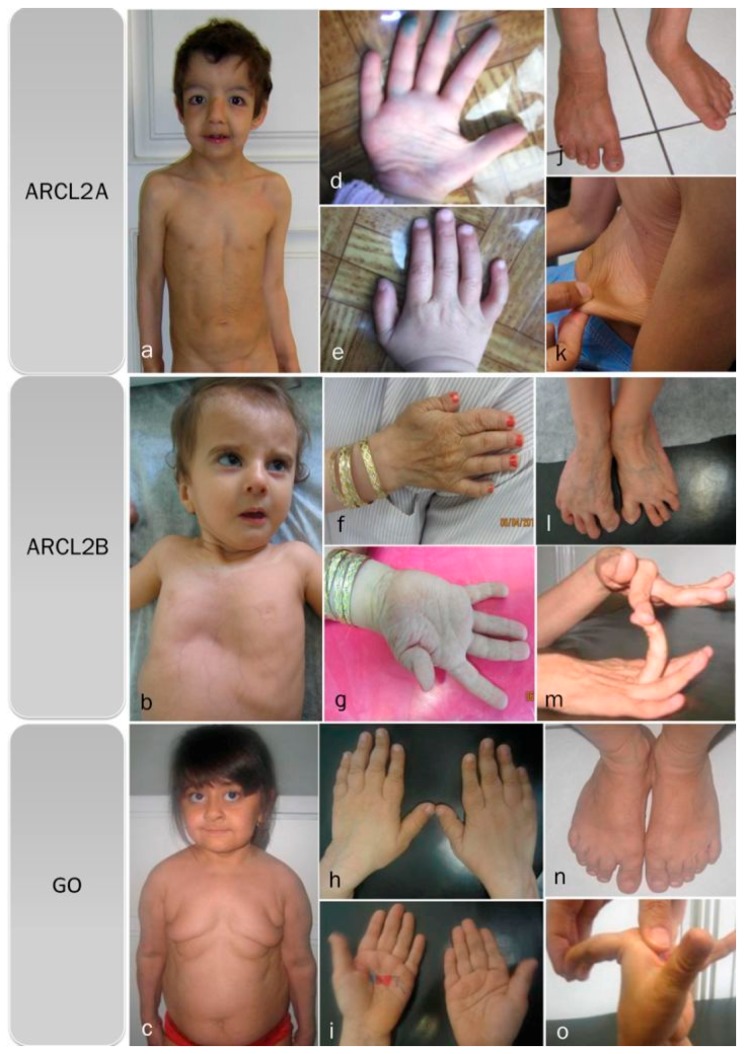
Overlapping clinical features of ARCL2A, ARCL2B, and GO cases. (**a**) Frontal view of patients with cutis laxa IIA, case 1; (**b**) frontal view of patient with ARCL2B case 6, note pectus excavatum, translucent skin, and visible veins; (**c**) frontal view of patient with GO case 7, note sagging and overfolding of skin; (**d**,**e**) note wrinkling of skin in the dorsum of hands and increased palmar creases in case 1; (**f**,**g**) adducted thumb seen in case 6; (**h**,**i**) wrinkling of skin and increased palmar creases in case 7; (**j**) flat feet in ARCL2A case 1; (**k**) cutis laxa in ARCL2A in case 2; (**l**) flat feet and increased wrinkling in dorsum of feet in ARCL2B patient case 3; (**m**) hyperlaxity of joints in ARCL2B patient case 3; (**n**) flat feet in GO case 7; and (**o**) hyperlaxity of joints in GO in case 7.

Cutis laxa, progeroid features, hyperextensible joints, inelastic wrinkled skin, and flat feet are features found in all three cutis laxa syndromes (Figure 1a–o) (Table 2). All of our patients, regardless of the cutis laxa syndrome, demonstrated flat feet however the frequency of feature has not been reported in the literature. Hypotonia and congenital hip dislocation are also found in all three conditions, but the severity and frequency of these findings varies. Short stature and open fontanel are common findings in cutis laxa types 2A and 2B, but occur less frequently in GO (Table 2); however, in our patients ¾ showed short stature. Osteopenia is characteristic of both ARCL2B and GO, though it is much more severe in GO and may even lead to pathologic fractures, especially of the vertebral column. We cannot comment on osteopenia in ARCL2A as it was not evaluated in the cases reported in the literature.

### 3.2. Facial Features

**Figure 2:**
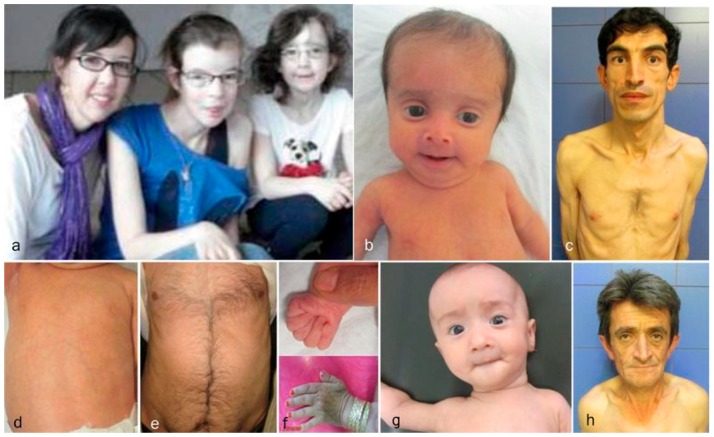
Comparison of aging in ARCL2A, ARCL2B, and GO. (**a**) Cutis laxa and facial dysmorphic features improves considerably in cutis laxa IIA as seen in three sisters affected with this condition; (**b**) the infant with ARCL2B has a triangular face (case 3); (**c**) The adult with ARCL2B has a long face with prognathism (case 5); (**d**) the trunk in patient with ARCL2B, the skin is translucent showing underlying vessels (case 3); (**e**) the trunk in the older patient shows improvement in translucency of skin (case 4); (**f**) the hand is tightly clenched in infancy in ARCL2B patient case 3 but can be easily opened as the child grows older; (**g**) infant with GO (case 9); (**h**) adult with GO (case 10); the GO patient has the least dysmorphic features and there is no dramatic change in facial features.

Each of the cutis laxa syndromes has distinct facial features. The ARCL2A patients demonstrate frontal bossing, large open fontanels, reverse V eyebrows, downslanting palpebral fissures, strabismus, a long philtrum and sagging cheeks with anteverted nares. The downslanting palpebral fissures and long philtrum are particularly indicative of ARCL2A and may help in establishing the diagnosis. The ARCL2B patients present during infancy and childhood with thin triangular faces, broad foreheads, a thin nose, and thin skin. Patients with this syndrome usually lack the sagging cheeks commonly seen in infants with ARCL2A and GO (Figure 2b). Though there are only a few adults with ARCL2B reported in the literature, the evidence supports that in adulthood, this face is no longer triangular but, rather, becomes long, with a prominent chin [15,17] (Figure 2c). Patient with GO demonstrate maxillary hypoplasia, sagging cheeks, and oblique furrowing, which extends from the outer canthus to the lateral border of the supraorbital ridge. This characteristic is generally more prominent in infancy and childhood (Figure 2g,h).

### 3.3. Progeroid Features

**Figure 3:**
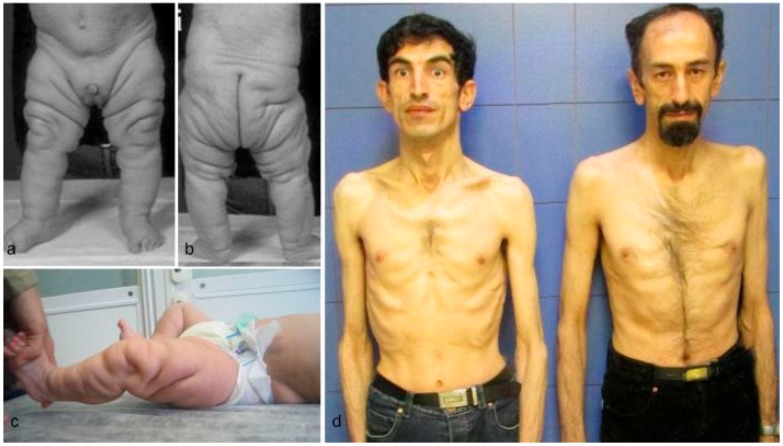
(**a**,**b**) Abnormal fat distribution seen in cutis laxa IIA; (**c**) it may rarely be seen in GO patients; and (**d**) note lipodystrophy in cases 4 and 5.

Clinical findings vary as the children with these cutis laxa syndromes grow older. At birth, the ARCL2B patients are usually very small with many having intrauterine growth retardation. The adult ARCL2B patient, however, is not particularly short but usually has a low weight and exhibits lipodystrophy (Figure 2c). ARCL2B infants have very thin skin, visible veins over thorax and clenched hands, but these symptoms improve considerably as the patient grows older (Figure 2d–f). Infants with GO usually have normal birth weights and lengths. The progeroid features improve in the majority of patients as the child grows older and reaches adult age. This progression is most obvious in ARCL2A (Figure 2a) [5,7,17]. Contrary to the improvement in the progeroid features, the abnormal fat distribution seen in ARCL2A worsens with age (Figure 3a–c) [4].

Based on the patients examined in this study, it seems that joint hyperlaxity improves as the patients reach adulthood. Hyperlaxity was seen in all of our child and infant cases, but of the three adult cases reported in this manuscript (two ARCL2B patients and one GO patient), all had very mild to absent joint hyperlaxity.

### 3.4. Neurological Features

**Figure 4:**
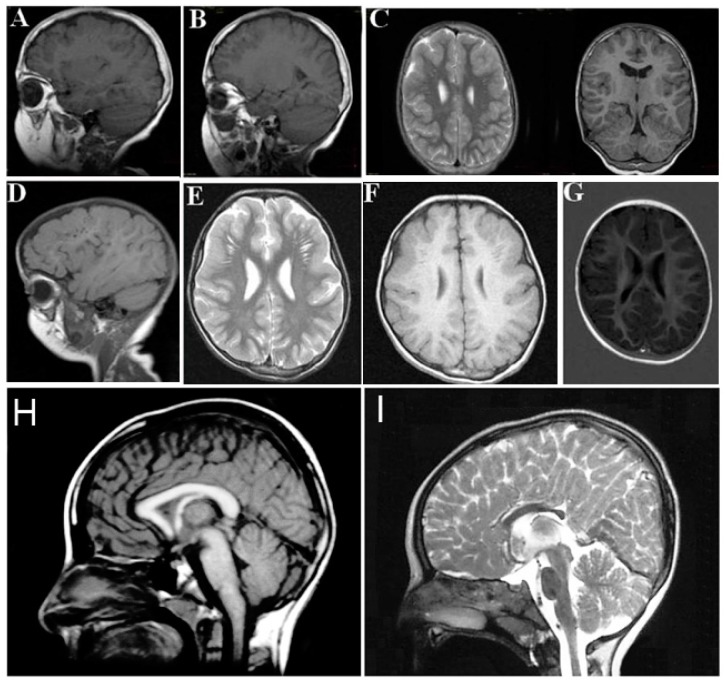
(**A**,**B**,**D**) demonstrate frontal and frontotemporal cobblestone-like brain dysgenesis on lateral T1 weighed images in three patients with cutis laxa IIA. Note the enlarged Virchow space under the abnormally-developed brain region; (**C**,**E**,**F**) shows sagittal images of fronto-temporal cobblestone-like brain dysgenesis on T2 and T1 weighted images in the same patients; (**G**) sagittal image showing mild, frontal cobblestone-like brain dysgenesis; (**I**) control showing normal-sized corpus callosum; and (**H**) agenesis of the corpus callosum in a cutis laxa IIB patient.

The neurological findings common to ARCL2A and ARCL2B patients are intellectual disability, hypotonia, psychomotor retardation, microcephaly, and seizures. Patients with GO, however, only demonstrate hypotonia if they demonstrate any neurological findings at all. Brain MRI imaging would be expected to differentiate between ARCL2A and ARCL2B based on the presence of cobblestone dysgenesis in ARCL2A compared to dysgenesis or agenesis of corpus callosum in cutis laxa IIB (Figure 4A–H) [2,5]. Brain MRI was not performed as part of this study and these features were not evaluated in our reported patients. Additionally, athetoid movements can occasionally be seen in ARCL2B and hearing loss has been reported in ARCL2A.

Microcephaly may be congenital or may develop during the course of the disease in ARCL2A [5]. Intellectual disability has been reported in ARCL2B but with the following exceptions: one large family with a complete deletion of *PYCR1* gene [16], in another patient with the c.138+1G>A mutation [2], and in three patients with missense alterations not residing in exons 4 to 6 [17]. The frequency of intellectual disability in ARCL2A is 78% based on all the reported cases in the literature. Almost all of the patients with GO that have been reported in the literature have normal cognition (94%).

### 3.5. Discriminative Features in ARCL2A

The features unique to patients with ARCL2A are abnormal isoelectric focusing, downslanting palpebral fissures, and a long philtrum.

### 3.6. Discriminative Features in ARCL2B

Facial features specific for ARCL2B are a thin triangular face with pinched nose in infancy and childhood that progresses to a long face with a prominent chin in adulthood. Severe wrinkling of the dorsum of the hands and feet is also more prominent in ARCL2B compared to ARCL2A and GO. Though not seen in all patients with cutis laxa IIB, wormian bones, athetoid movements, lipodystrophy, cataracts and corneal clouding are highly specific for ARCL2B. These features are either not seen or very rarely seen in the other two cutis laxa syndromes [17].

### 3.7. Discriminative Features in GO

Patients with GO have normal mentation, whereas the majority of the patients with ARCL2A and ARCL2B have variable degrees of intellectual disability. While patients with ARCL2B may manifest osteopenia, osteopenia that is severe enough to cause pathologic fractures is more characteristic of GO. Facial features specific to GO are maxillary hypoplasia and oblique furrowing from the outer canthus to the lateral border of the supraorbital ridge.

## 4. Conclusions

In conclusion, we believe that in spite of great similarity in ARCL2A, ARCL2B, and GO syndromes, there are discriminative features unique to each condition that can guide accurate clinical diagnosis of cutis laxa syndrome subtypes, so that we were able to correctly make the clinical diagnosis. Sanger sequencing of the candidate gene would be much more cost effective to the general approach of next-generation sequencing cutis laxa panels.

## Figures and Tables

**Table 1:** Comparison of clinical features of cutis laxa IIA, IIB, and GO syndrome, and 10 patients with these cutis laxa conditions.

Diagnosis	Cutis laxa IIA	Family I Case 1	Family II Case 2 *	Cutis laxa IIB	Family III Case 3	FamilyIV Case 4	Family IV Case 5	Family V Case 6	GO	Family VI Case 7	Family VII Case 8	Family VIII Case 9	Family IX Case 10
		ARCL2A	ARCL 2A		ARCL2B	ARCL2B	ARCL2B	ARCL2B		GO	GO	GO	GO
Age at examination		4 years	12 years		14 months	35 years	36 years	1 year		9 years	3 years	6 months	40 years
Sex		F	M		F	M	M	F		F	M	F	M
Intra-uterine growth retardation	+	+	+	+	+	+	+	+	-	-	-	+	NE
height		108 cm 95th centile	140 cm 10th centile		75 cm <50th centile	173 cm <50th centile	175 cm <50th centile	68 cm <25th centile		115 cm <3rd centile	69 cm <3rd centile	65 cm 50th centile	158 cm <3rd centile
weight		12 kg <3rd centile	27 Kg <3rd centile		6.8 kg <3rd centile	40 kg <3rd centile	45 <3rd centile	6.2 kg <3rd centile		24 kg <25th centile	7.4 kg <3rd centile	6.4 kg <50th centile	50 kg <3rd centile
Head Circumference		47.5 cm <50th centile	51 cm <50th centile		42 cm <2nd centile	53 cm <50th centile	54.5 <50th centile	40 cm <2nd centile		50 cm <50th centile	42 cm <2nd centile	41 cm <25th centile	57.5 cm <98th centile
Facial features													
Aged appearance	+	+	+/−	+	+	+	+	+	+	+	+	+	+
Persistent open fontanel	+	NE	+	+	-	NE	NE	+	−	NE	−	−	NE
Bossing forehead/broad forehead	+	−	−	+	+	−	−	+	−	−	+	−	−
Downslanting palpebral fissures	+	+	+	−	−	−	−	−	−	−	−	−	−
Reverse V eyebrows	+	+	+	−	−	−	−	−	−	−	−	−	−
Long philtrum	+	+	+	−	−	−	−	−	−	−	−	−	−
Anteverted nares	+	+	+	−	−	−	−	−	−	−	−	−	−
Blue sclera	+	−	−	+	+	−	−	+	−	−	−	−	−
Triangular face		−	−	+	+	−	+	+	−	−	−	−	−
Thin nose		−	−	+	+	+	+	+	−	−	−	−	−
Long face		−	−	+ **	−	+	+	−	−	−	−	−	−
prognathism		−	−	+ **	−	+	+	−	−	−	−	−	−
Maxillary hypoplasia		−	−	−	−	−	−	−	+	+	+	−	+
Oblique furrowing extending from the outer canthus to the lateral border of the supraorbital ridge		−	−	−	−	−	−	−	+	−	+	+	−
Drooping/sagging cheeks	+	+	−	−	−	−	−	−	+	+	+	+/−	−
Neurological abnormalities													
Developmental delay	+	+	+	+	+	+	+	+	−	−	−	−	−
Intellectual disability	+	+	Very mild	+	+	+	+	+	−	−	−	−	−
Microcephaly		−	−	+	+	−	−	+	−	−	−	−	−
Hypotonia	+	+	+ at birth	+	+	−	−	+	−	−	−	−	−
Eye abnormalities								−					
Myopia	+	+	−	−	NE	−	−	−	−	−	−	−	−
Strabismus	+	−	+	−	+	−	−	−	−	−	−	−	−
Skeleton													
Pectus excavatum	+	−	+	+	−	−	−	+	−	−	−	−	−
Osteopenia	+	+	−	+	+	NE	NE	NE	+	+	+	+	+
Fractures		−	−	+/−	−	−	−	−	+	+	−	−	+
Congenital hip dislocation	+	+	−	+	−	−	−	+	+/−	−	−	+	−
Skin & joints													
Hyperextensibility of joints	+	+	+	+	+	+/−	+/−	+	+	+	+	+	−
Dislocations		−	−		+	-	−	+	−	−	−	−	−
Adducted thumbs		−	−	+	+	+	+	+	−	+	−	−	−
Clenched hands		−	−	+	+	−	−	+	−	+	−	−	−
Increased palmar creases		+	+		+	+	+	+		+	+	+	+
Wrinkled skin	+	+	+	+	+	+	+	+	+	+	+	+	+
Severe wrinkling of skin on dorsum of hands and feet		−	−	+	+	+	+	+	−	+	−	−	−
Visible veins on the chest		−	−	+	+	+	+	+	−	−	+	−	−
Inguinal hernia	+	−	+	+	−	−	−	−	−	−	+	−	−
Umbilical hernia		−	−	+	−	−	−	+	−	−	−	+	−
Lipodystrophy		−	−	+	−	+	+	+	−	−	−	−	−
Flat feet	+	−	+	+		+	+	+	+	−	−	−	+
Causative *gene* Mutation cDNA Mutation protein	*ATP6V0A2*	c.2255C>T homozygous mutation in *ATP6V0A2* novel	c.754delT homozygous mutation in *ATP6V0A2* ^22^		c.797 G>A homozygous mutation in *PYCR1* gene ^2,3^	c. 355C>T homozygous mutation in *PYCR1* gene ^2^	c. 355C>T homozygous mutation in *PYCR1* gene ^2^	c.572G>A homozygous mutation in *PYCR1* gene ^15^		c.-1_1GA>CT homozygous mutation in *GORAB* gene ^21^	c.-1_1GA>CT homozygous mutation in *GORAB* gene	c.391C>T homozygous mutation in *GORAB* gene novel	c.75_76delinsCT homozygous mutation in *GORAB* gene *^21^*
other		Glycosylation abnormalitites	Glycosylation abnoramalities					Cleft palate				Small ASD	

* Case 2 was previously reported by Gardeitchik et al., 2014 [22]; ** Case 6 was previously reported by Kretz et al., 2011 [15]; *** present in adulthood + present; − absent; F, Female; M, Male; NE, Not evaluated.

**Table 2:** Comparison of clinical features of cases with ARCL2A, ARCL2B, and GO in present cases and cases from the literature. NE, not evaluated.

Clinical Features	ARCL2A Total Cases from Literature		ARCL2A Present Cases	ARCL2B Total Cases from the Literature		ARCL2B Present Cases	GO Total Cases from the Literature		GO Present Cases
	Number of Positive Cases from Number of Evaluated Cases	Frequency (%)		Number of Positive Cases from Number of Evaluated Cases	Frequency (%)		Number of Positive Cases From Number of Evaluated Cases	Frequency (%)	
Intra-Uterine Growth Retardation	7/17	41%	2/2	78/83	94%	4/4	0/9	0%	1/3
Postnatal Growth Delay	10/24	42%	0/2	43/64	67%	4/4	8/52	15%	3/4
Aged Appearance	25/25	100%	2/2	89/87	98%	4/4	49/53	92%	4/4
Persistent open Fontanel	53/58	91%	1/1	30/40	75%	1/2	2/12	17%	0/2
Blue sclera	2/4	50%	1/2	23/48	48%	2/2	1/19	5%	0/4
Intellectual Disability	45/58	78%	2/2	86/92	93%	4/4	3/53	6%	0/4
Microcephaly	33/47	70%	0/2	32/49	65%	2/4	1/53	2%	1/4
Hypotonia	29/35	83%	2/2	33/46	72%	2/4	13/38	34%	0/4
Seizures	13/53	25%	0/2	14/62	23%	0/4	1/49	2%	0/4
Athetoid/Dystonic Movements	1/15	6%	0/2	17/85	20%	0/4	0/49	0%	0/4
Corpus Callosum Dysgenesis	2/20	10%	NE	26/53	49%	NE	0/3	0%	NE
Pachygryria	30/46	65%	NE	0/53	0%	NE	0/3	0%	NE
Strabismus	21/48	44%	1/2	17/48	35%	1/4	0/3	0%	0/4
Cataract/corneal clouding	1/38	3%	0/2	13/88	15%	0/4	0/3	0%	0/4
Osteopenia	NE	NE	1/2	38/52	73%	1/1	50/52	96%	4/4
Fractures	5/55	9%	0/2	3/23	13%	0/4	39/53	74%	2/4
Congenital hip dislocation	9/15	60%	1/2	53/86	62%	1/4	36/53	68%	1/4
Wormian bonses	NE	NE	0/2	20/32	63%	NE	15/36	42%	NE
Hyperextensibility of joints	40/46	87%	2/2	75/79	95%	4/4	45/46	98%	3/4
Dislocations	0/7	0%	0/2	2/8	25%	2/4	NE	NE	0/4
Adducted thumbs	0/5	0%	0/2	28/45	62%	4/4	NE	NE	1/4
Wrinkled skin	60/60	100%	2/2	93/93	100%	4/4	53/53	100%	4/4
Visible veins on the chest	NE	NE	0/2	46/53	87%	4/4	9/43	21%	0/4
Hernia	17/36	47%	1/2	33/84	39%	2/4	1/43	2%	2/4
Flat feet	1/3	33%	1/2	5/7	71%	3/4	NE	NE	1/4

## Appendix A

**Table A1:** Clinical features in autosomal recessive cutis laxa 2A patients reported in the literature.

Clinical Features	Morava et al., 2008 [4]	Van Maldergem et al., 2008 [7]	Hucthagowder et al., 2009 [6]	Fischer et al., 2012 [23]	Greally et al., 2014 [24]	Gardeitchik et al. 2014 [22]	Bahena-Bahena et al., 2014 [25]	Ritelli et al., 2014 [26]	Goyal et al., 2015 [27]	Cohen et al., 2016 [28]	Total
Diagnosis	ARCL2A	ARCL2A	ARCL2A	ARCL2A	ARCL2A	ARCL2A	ARCL2A	ARCL2A	ARCL 2A	ARCL2A	
Intra-uterine growth retardation	2/10	NE	NE	NE	3/3	NE	NE	+	+	0/2	5/7
Postnatal growth delay	NE	NE	7/17	NE	0/3	NE	2/2	-	+	NE	10/24
Aged appearance	10/10	11/11	NE	NE	NE	NE	2/2	+	+	NE	4/4
Persistent open fontanel	10/10	11/11	16/17	10/12	3/3	NE	2/2	+	NE	0/2	32/37
Blue sclera	NE	NE	NE	NE	NE	NE	NE	+	+	0/2	2/4
Intellectual disability	8/10	11/11	12/17	6/11	3/3	NE	2/2	-	+	2/2	27/37
Microcephaly	7/10	4/11	14/17	NE	3/3	NE	2/2	-	+	2/2	23/26
Hypotonia	8/10	11/11	NE	NE	1/3	6/6	2/2	-	NE	½	10/13
seizures	3/10	5/11	1/17	NE	1/3	2/6	0/2	-	-	1/2	5/30
Athetoid/dystonic movements	NE	NE	NE	NE	0/3	1/6	0/2	-	-	0/2	1/15
Corpus callosum dysgenesis	NE	1/11	NE	NE	1/3	0/4	NE	NE	NE	0/2	1/9
Pachygryria/cobblestone like dysgenesis	4/10	8/11	9/15	1/1	2/3	4/4	NE	NE	NE	2/2	18/25
Strabismus	7/10	0/11	13/17	NE	NE	2/6	NE	-	-	1/2	16/27
Cataract/corneal clouding	NE	0/11	0/17	NE	NE	1/6	NE	-	-	0/2	0/27
Osteopenia	NE	NE	NE	NE	NE	NE	NE	NE	-	NE	NE
Fractures	NE	4/11	0/17	0/12	0/3	0/6	0/2	+	-	0/2	1/44
Congenital hip dislocation	NE	8/8	NE	NE	0/3	NE	NE	-	+	0/2	1/7
Wormian bonses	NE	NE	NE	NE	NE	NE	NE	NE	NE	NE	NE
Hyperextensibility of joints	9/10	NE	14/17	10/12	3/3	NE	2/2	NE	NE	2/2	31/36
Dislocations	NE	NE	NE	NE	0/3	NE	NE	-	-	0/2	0/7
Adducted thumbs	NE	NE	NE	NE	0/3	NE	NE	NE	NE	0/2	0/5
Wrinkled skin	10/10	11/11	17/17	13/13	3/3	NE	2/2	+	+	2/2	39/39
Visible veins on the chest	NE	NE	NE	NE	NE	NE	NE	NE	-	NE	NE
hernia	NE	6/11	7/16	NE	2/3	NE	½	+	-	0/2	11/25
Flat feet	NE	NE	NE	NE	NE	NE	NE	+	NE	0/2	1/3

ARCL2A, autosomal recessive cutis laxa 2A; NE, not evaluated.

**Table A2:** Clinical features in autosomal recessive cutis laxa 2B patients reported in the literature.

Clinical Features	Reversade et al., 2009 [2]	Guernsey et al., 2009 [3]	Yildirim et al., 2010 [16]	Lin et al., 2011 [29]	Kretz et al., 2011 [15]	Dimopoulou et al., 2013 [17]	Nouri et al., 2013 [30]	Scherrer et al., 2013 [31]	Gardeitchik et al. 2014 [22]	Rahmati et al., 2015 [32]	Goyal et al., 2015 [27]	Alazami et al., 2016 [33]	Vahidnezhed et al., 2016 [34]	Total
Diagnosis	ARCL2B	ARCL2B	ARCL2B	ARCL2B	ARCL2B	ARCL2B	ARCL2B	ARCL2B	ARCL2B	ARCL2B	ARCL2B	ARCL2B	ARCL2B	
Intra-uterine growth retardation	29/31	5/5	4/4	2/2	6/6	23/25	-	1/1	NE	+	+	+	5/5	69/83
Postnatal growth delay	4/13	5/5	0/4	1/2	6/6	21/28	NE	3/3	NE	+	+	+	NE	43/64
Aged appearance	31/31	5/5	4/4	2/2	6/6	29/30	+	3/3	NE	+	+	+	5/5	86/87
Persistent open fontanel	3/6	3/3	2/3	1/2	NE	14/19	NE	3/3	NE	-	+	+	2/2	31/40
Blue sclera	NE	4/5	NE	1/2	1/6	13/30	NE	NE	NE	+	+	-	2/2	23/47
Intellectual disability	34/35	5/5	4/4	2/2	3/6	27/29	+	3/3	NE	NE	+	+	5/5	86/92
Microcephaly	NE	4/5	2/4	2/2	4/5	19/27	NE	0/3	NE	-	+	-	NE	32/47
Hypotonia	NE	0/3	NE	0/2	NE	25/30	NE	2/3	5/5	+	-	-	NE	33/44
seizures	NE	NE	2/4	0/2	0/6	3/33	+	0/3	5/5	-	-	+	2/5	14/61
Athetoid /dystonic movements	4/35	0/5	0/4	½	NE	9/28	NE	0/3	3/5	-	-	-	NE	17/85
Corpus callosum dysgenesis	15/21	2/3	1/4	0/2	NE	4/13	+	NE	2/5	-	NE	+	0/2	26/53
pachygryria	0/21	0/3	0/4	0/2	NE	0/13	-	NE	0/5	-	NE	-	0/2	0/53
Strabismus	NE	NE	NE	½	1/6	12/27	NE	0/3	2/5	-	-	-	½	17/43
Cataract/corneal clouding	4/35	0/5	NE	0/2	0/6	4/27	NE	0/3	4/5	-	-	+	0/2	12/88
Osteopenia	12/18	0/1	4/4	0/2	3/3	14/16	NE	3/3	NE	-	-	-	2/2	38/52
Fractures	NE	0/3	0/4	0/2	1/6	NE	NE	2/3	NE	-	-	-	0/2	3/23
Congenital hip dislocation	19/34	2/4	¼	½	4/6	15/25	NE	3/3	NE	+	+	+	5/5	43/86
Wormian bonses	6/9	1/2	4/4	0/2	1/1	8/12	NE	NE	NE	-	NE	-	NE	20/32
Hyperextensibility of joints	35/35	NE	4/4	2/2	4/6	24/26	NE	3/3	NE	NE	NE	+	2/2	75/79
Dislocations	NE	NE	NE	0/2	NE	NE	NE	0/3	NE	NE	NE	-	2/2	2/8
Adducted thumbs	NE	3/3	4/4	½	4/6	13/26	NE	2/3	NE	NE	NE	+	NE	24/45
Wrinkled skin	35/35	3/3	4/4	2/2	6/6	31/31	+	3/3	NE	+	+	+	5/5	93/93
Visible veins on the chest	NE	5/5	4/4	2/2	6/6	24/31	NE	NE	NE	+	+	+	2/2	46/53
hernia	17/35	2/3	1/4	0/2	0/6	10/28	NE	3/3	NE	-	-	-	NE	33/81
Flat feet	NE	NE	NE	NE	5/6	NE	NE	NE	NE	NE	NE	-	NE	5/7

ARCL2B, autosomal recessive cutis laxa 2B; NE, not evaluated.

**Table A3:** Clinical features in Geroderma Osteodysplastica patients reported in the literature.

Clinical Features	Newman et al., 2008 [35]	Al-Dosari et al., 2009 [19]	Gardeitchik et al., 2014 [22]	Alazami et al., 2016 [33]	Total
Diagnosis	GO	GO	GO	GO	GO
Intra-uterine growth retardation	NE	NE	NE	NE	NE
Postnatal growth delay	5/9	1/7	NE	2/27	8/43
Aged appearance	10/10	7/7	NE	23/27	40/44
Persistent open fontanel	NE	1/7	NE	NE	1/7
Blue sclera	1/10	NE	NE	NE	1/10
Intellectual disability	0/10	0/7	NE	0/27	0/44
Microcephaly	0/10	0/7	NE	0/27	0/44
Hypotonia	8/8	NE	3/3	2/27	13/38
seizures	0/10	NE	1/3	0/27	1/40
Athetoid/dystonic movements	0/10	NE	0/3	0/27	0/40
Corpus callosum dysgenesis	NE	NE	0/3	NE	0/3
pachygryria	NE	NE	0/3	NE	0/3
Strabismus	NE	NE	0/3	NE	0/3
Cataract/corneal clouding	NE	NE	0/3	NE	0/3
Osteopenia	10/10	6/7	NE	26/27	42/44
Fractures	9/10	5/7	NE	17/27	31/44
Congenital hip dislocation	4/10	2/7	NE	25/27	31/44
Wormian bonses	NE	NE	NE	15/27	15/27
Hyperextensibility of joints	10/10	NE	NE	26/27	36/37
Dislocations	NE	NE	NE	NE	NE
Adducted thumbs	NE	NE	NE	NE	NE
Wrinkled skin	10/10	7/7	NE	27/27	44/44
Visible veins on the chest	0/10	NE	NE	0/27	0/34
hernia	0/10	NE	NE	0/27	0/34
Flat feet	NE	NE	NE	NE	NE

NE, not evaluated.
